# Risk factors, host response and outcome of hypothermic sepsis

**DOI:** 10.1186/s13054-016-1510-3

**Published:** 2016-10-14

**Authors:** Maryse A. Wiewel, Matthew B. Harmon, Lonneke A. van Vught, Brendon P. Scicluna, Arie J. Hoogendijk, Janneke Horn, Aeilko H. Zwinderman, Olaf L. Cremer, Marc J. Bonten, Marcus J. Schultz, Tom van der Poll, Nicole P. Juffermans, W. Joost Wiersinga

**Affiliations:** 1 Center for Experimental and Molecular Medicine, Academic Medical Center, University of Amsterdam, Amsterdam, The Netherlands; 2 Department of Intensive Care, Academic Medical Center, University of Amsterdam, Amsterdam, The Netherlands; 3 Clinical Epidemiology Biostatistics and Bioinformatics, Academic Medical Center, University of Amsterdam, Amsterdam, The Netherlands; 4 Division of Infectious Diseases, University Medical Center Utrecht, Utrecht, The Netherlands; 5 Department of Intensive Care Medicine, University Medical Center Utrecht, Utrecht, The Netherlands; 6 Department of Medical Microbiology, University Medical Center Utrecht, Utrecht, The Netherlands; 7 Julius Center for Health Sciences and Primary Care, University Medical Center Utrecht, Utrecht, The Netherlands

**Keywords:** Hypothermia, Sepsis, Mortality, Risk factors, Host response, Fractalkine

## Abstract

**Background:**

Hypothermia is associated with adverse outcome in patients with sepsis. The objective of this study was to characterize the host immune response in patients with hypothermic sepsis in order to determine if an excessive anti-inflammatory response could explain immunosuppression and adverse outcome. Markers of endothelial activation and integrity were also measured to explore potential alternative mechanisms of hypothermia. Finally we studied risk factors for hypothermia in an attempt to find new clues to the etiology of hypothermia in sepsis.

**Methods:**

Consecutive patients diagnosed with sepsis within 24 hours after admission to ICUs in two tertiary hospitals in the Netherlands were included in the study (*n* = 525). Hypothermia was defined as body temperature below 36 °C in the first 24 h of ICU admission.

**Results:**

Hypothermia was identified in 186 patients and was independently associated with mortality. Levels of proinflammatory and anti-inflammatory cytokines were not different between groups. Hypothermia was also not associated with an altered response to ex vivo stimulation with lipopolysaccharide in a subset of 15 patients. Risk factors for hypothermia included low body mass index, hypertension and chronic cardiovascular insufficiency. Levels of the endothelial activation marker fractalkine were increased during the first 4 days of ICU stay.

**Conclusions:**

Hypothermia during sepsis is independently associated with mortality, which cannot be attributed to alterations in the host immune responses that were measured in this study. Given that risk factors for hypothermic sepsis are mainly cardiovascular and that the endothelial activation marker fractalkine increased in hypothermia, these findings may suggest that vascular dysfunction plays a role in hypothermic sepsis.

**Electronic supplementary material:**

The online version of this article (doi:10.1186/s13054-016-1510-3) contains supplementary material, which is available to authorized users.

## Background

Sepsis is the consequence of a dysregulated immune response to infection, involving both proinflammatory and anti-inflammatory components, and a highly activated endothelium, resulting in increased vascular permeability, organ failure and shock [1]. Fever and hypothermia are both hallmark characteristics of sepsis [2]. Hypothermia is observed in 9 − 35 % of patients with sepsis [3, 4]. Whereas fever is generally considered beneficial for patients, hypothermia is independently associated with increased mortality [4]. However the etiology of hypothermia during sepsis is poorly understood.

Studies attempting to elucidate the hypothermic response in sepsis have focused on a hypothesized lack of proinflammatory cytokines, in particular interleukin (IL)-6 and tumor necrosis factor (TNF)-α, which are the main mediators of fever. However, these studies could not demonstrate that a depression of the proinflammatory response is associated with hypothermia in sepsis [5, 6]. Anti-inflammatory cytokines such as IL-10, which possess antipyretic properties in humans [7] and animals [8], have not been studied before in hypothermic patients with sepsis. An excessive anti-inflammatory response could potentially explain hypothermia [9] and associated lymphopenia, which was recently found in hypothermic patients with sepsis [10].

Other mechanisms have yet to be explored in hypothermic patients with sepsis. Endothelial dysfunction could also play a role in the development of hypothermia, as generalized peripheral vasodilation and loss of endothelial integrity during sepsis may result in heat loss by hampering the body’s ability to regulate its core temperature [11, 12].

The aim of this current prospective observational study was to determine if hypothermia is independently associated with 90-day mortality. We subsequently characterized the host immune response in patients with hypothermic sepsis, by determining both proinflammatory and anti-inflammatory cytokines, and whole blood ex vivo responsiveness to lipopolysaccharide (LPS). We also measured markers of endothelial activation and integrity. Finally, we studied risk factors for hypothermic sepsis in an attempt to find potential new insights into the etiology of hypothermia in sepsis. Understanding the etiology of hypothermic sepsis may contribute to the identification of potential targets for future interventions.

## Methods

### Study design, patients and definitions

From January 2011 through July 2013, consecutive patients presenting to the mixed intensive care units (ICU) of two Dutch tertiary teaching hospitals (Academic Medical Center in Amsterdam and University Medical Center in Utrecht) were included. Medical Ethical Committees of both centers approved an opt-out consent method (IRB no.10-056C). Data and plasma samples were prospectively collected as part of the Molecular Diagnosis and Risk Stratification of Sepsis (MARS) project (ClinicalTrials.gov identifier NCT01905033) [13, 14]. A group of trained investigators collected clinical data. The plausibility of infection was scored post hoc, and classified on a 4-point scale (none, possible, probable or definite) [15, 16], as described in detail previously [14]. Shock was defined as hypotension requiring treatment with vasopressors at a dose of 0.1 mcg/kg/min during at least 50 % of the day. Acute kidney injury (AKI) and acute lung injury (ALI) were scored using pre-set criteria [17, 18]. Blood samples were collected in EDTA tubes, centrifuged and stored until further analysis.

We selected patients with sepsis, diagnosed within 24 h of admission, defined as a having definite or probable infection [14], combined with at least one parameter of inflammatory dysfunction, hemodynamic dysfunction, organ dysfunction or deranged tissue perfusion (derived from the 2001 International Sepsis Definitions Conference [2]). Patients with immunodeficiency disorders, use of corticosteroids, immunosuppressive or antineoplastic drugs were excluded. To exclude iatrogenic hypothermia, readmitted patients, patients undergoing active cooling and patients transferred from another ICU or operating room (OR) were also excluded.

To control for body temperatures that may have been inadvertently entered in the database (i.e. a rectal sensor that has been displaced and is exposed to ambient temperature), patients with unreliable measurements of temperature (below 33 °C) were not included. Also, patients with a missing minimum temperature were not included. Temperature was measured using a rectal, nasal, inguinal or tympanic temperature probe. Core temperatures were used in preference to inguinal or tympanic measurements. The threshold for hypothermia was set at 36 °C, based on previously used cutoffs [10, 19]. Daily (at admission and at 6. a.m. thereafter) leftover EDTA coagulated plasma (obtained from blood drawn for patient care) was stored within 4 h at −80 °C. Samples were drawn prior to rewarming patients.

### Plasma biomarker measurements

TNF-α, IL-1β, IL-6, IL-8, IL-10, IL-13, soluble intercellular adhesion molecule (ICAM)-1, fractalkine and soluble E-selectin were measured using FlexSet cytometric bead arrays (BD Bioscience, San Jose, CA, USA) using FACSCalibur (Becton Dickenson, Franklin Lakes, NJ, USA). Angiopoietin-1 and angiopoietin-2 (R&D systems, Abingdon, UK) were measured by Luminex multiplex assay using BioPlex 200 (BioRad, Hercules, CA). Normal biomarker values were acquired from EDTA plasma from 27 age-matched and gender-matched healthy volunteers, from whom written informed consent was obtained.

The lower limits of detection for the immune assays were: 0.9 pg/mL for TNF-α, 1.3 pg/mL for IL-1β, 0.9 pg/mL for IL-6, 1.3 pg/mL for IL-8, 0.8 pg/mL for IL-10, 0.7 pg/mL for IL-13, 3.1 pg/mL for soluble E-selectin, 6.3 pg/mL for soluble ICAM-1, 4.0 pg/mL for fractalkine, 0.2 pg/mL for angiopoietin-1 and 1.8 pg/mL for angiopoietin-2.

### Whole blood stimulations

In a random subset of 15 patients, whole blood was stimulated ex vivo with LPS on day 1 of ICU admission, as previously described [20]. Heparin-anticoagulated blood was stimulated for 3 h at 37 °C in pyrogen-free RPMI 1640 (Life Technologies, Bleiswijk, the Netherlands) with or without 100 ng/mL ultrapure LPS (from *Escherichia coli* 0111:B4; InvivoGen, Toulouse, France). TNF-α and IL-1β were measured in supernatants using a cytometric bead array assay (BD Biosciences, San Jose, CA, USA). Cytokine release was calculated as the difference in cytokine levels in samples incubated with and without LPS. The medical ethical committee of the Academic Medical Center in Amsterdam gave ethical approval for the study (number NL34294.018.10). Written informed consent was obtained from all patients, or their legal representative, and from healthy volunteers.

### Statistical analysis

All analyses were performed in R (version 3.1.1). Student’s *t* test or the Wilcoxon rank-sum test, and the chi-square test were used to compare groups. To study factors independently associated with developing hypothermia, we performed multivariable logistic regression. Pre-ICU-admission patient characteristics that were deemed relevant or were associated with hypothermia in univariate analysis (*P* < 0.2) were included in the model. A backward selection procedure using the Akaike information criterion (AIC) including 1000 bootstrap replicates was applied (R-package “rms”) to identify risk factors for hypothermia. Age was forced into the model because we considered it an important confounder for all factors incorporated in the model, and because it is associated with an altered temperature response [4, 21].

Multivariable logistic regression was used to establish the independent association between hypothermia and 90-day mortality. The Acute Physiology and Chronic Health Evaluation (APACHE) IV score was included in the model to adjust for severity of disease at ICU admission. Age, body mass index (BMI), admission type and source of infection were a priori considered potential clinically relevant confounders. Next, risk factors for hypothermia from logistic regression analyses were investigated as confounders of mortality. Significant variables were retained in the model, based on 10 % change in estimate. In order to determine whether hypothermia was associated with biomarker response irrespective of severity of disease, hypothermic patients were 1:1 matched to nonhypothermic patients according to APACHE IV scores, using “optimal matching” with R-package “MatchIt”. *P* < 0.05 was considered statistically significant.

## Results

### Epidemiology of hypothermic sepsis

The selection of study patients is presented in Additional file 1: Figure S1. From a total of 525 patients, 186 patients (35.4 %) were hypothermic during the first 24 h of admission. Patient characteristics are shown in Table 1. Mean body temperature in the first 24 h was significantly lower in hypothermic versus nonhypothermic patients (median 36.3 °C and 37.3 °C respectively). Mean age in hypothermic patients was significantly higher and BMI was lower. Hypothermic patients suffered more frequently from cardiovascular disease including chronic cardiovascular insufficiency, hypertension and cerebrovascular disease. Hypothermic patients were most often admitted from the emergency department. Also, a significantly higher proportion of patients with hypothermia had a urinary tract infection. We observed no differences in causative organisms (Additional file 1: Table S1). Hypothermic patients were more seriously ill, as reflected by higher APACHE IV and Sequential Organ Failure Assessment (SOFA) scores and increased incidence of AKI (and requirement of renal replacement therapy). In line with this, patients with hypothermia had higher maximum white blood cell counts, longer prothrombin times and increased creatinine and lactate levels.

**Table 1:** Baseline characteristics of sepsis patients with and without hypothermia during the first 24 h of admission

	Hypothermia	No hypothermia	*P*
	*N* = 186	*N* = 339	
Demographics			
Age, years, mean (SD)	65.0 (13.8)	61.1 (15.6)	0.004
Gender, male, *n* (%)	114 (61.3)	206 (60.8)	0.94
BMI, kg/m^2^, mean (SD)	25.6 (5.7)	26.7 (6.7)	0 .04
Comorbidities			
Charlson score, median (IQR)	5 (3–6)	4 (2–6)	0.01
Cerebrovascular disease, *n* (%)	28 (15.1)	27 (8)	0.01
Chronic cardiovascular insufficiency, *n* (%)	13 (7)	7 (2.1)	0.009
Chronic renal insufficiency, *n* (%)	26 (14)	32 (9.4)	0.15
Congestive heart failure, *n* (%)	8 (4.3)	17 (5)	0.84
COPD, *n* (%)	29 (15.6)	61 (18)	0.55
Diabetes mellitus, *n* (%)	42 (22.6)	67 (19.8)	0.51
Hypertension, *n* (%)	73 (39.2)	87 (25.7)	0.003
Liver cirrhosis, *n* (%)	7 (3.8)	6 (1.8)	0.23
Peripheral vascular disease, *n* (%)	25 (13.4)	44 (13)	0.90
Admission			
Admission type, medical, *n* (%) (%)	163 (87.6)	298 (87.9)	0.55
Admission origin, emergency department, *n* (%) (%)	74 (39.8)	99 (29.2)	0.04
medium care, *n* (%)	26 (14)	47 (13.9)	
ward, *n* (%)	86 (46.2)	193 (56.9)	
Site of infection			
Pulmonary, *n* (%)	79 (42.5)	161 (47.5)	0.28
Abdominal, *n* (%)	29 (15.6)	65 (19.2)	0.34
Urinary tract, *n* (%)	32 (17.2)	33 (9.7)	0.02
Other, *n* (%)	18 (9.7)	44 (13)	0.31
Co-infection, *n* (%)	28 (15.1)	36 (10.6)	0.15
Severity of disease first 24 h			
Mean temperature first 6 h, median (IQR)	36.1 (35.4–37)	37.2 (36.5–38)	<0.0001
Mean temperature first 24 h, median (IQR)	36.3 (35.8–36.9)	37.3 (36.8–37.8)	<0.0001
APACHE IV score, median (IQR)^a^	82 (67–103)	71 (58–86)	<0.0001
SOFA score, median (IQR)^b^	8 (5–10)	7 (4–8)	<0.001
Acute kidney injury, *n* (%)	92 (49.5)	118 (34.8)	0.002
Renal replacement therapy, *n* (%)	32 (17.1)	21 (6.2)	<0.001
Acute lung injury, *n* (%)	49 (26.3)	99 (29.2)	0.57
Shock, *n* (%)	74 (39.8)	106 (31.3)	0.06
Clinical laboratory parameters first 24 h			
WBC count max. (×10^9/L), median (IQR)	16.1 (10.9–25.5)	14.9 (10–19.2)	0.02
WBC count min. (×10^9/L), median (IQR)	12.6 (7.1–19.1)	12.2 (7.7–16.2)	0.25
Platelets min. (×10^9/L), median (IQR)	189 (120–264)	199 (131–283)	0.27
Lactate max. (mmol/L), median (IQR)	3.2 (1.6–6.5)	2.5 (1.6–4.1)	0.009
Prothrombin time max. (s), median (IQR)	16.5 (14.1–20.7)	15 (12.6–18.2)	0.0001
Creatinin max. (μmol/L), median (IQR)	121 (80–209)	97 (68–162)	<0.001
C-reactive protein (mg/L), median (IQR)	146 (82–258)	174 (98–263)	0.25

^a^Temperature not included in score. ^b^Central nervous system not included in score due to large number of sedated patients. *APACHE* Acute Physiology And Chronic Health Evaluation, *COPD* chronic obstructive pulmonary disease, *IQR* interquartile range, *SD* standard deviation, *SOFA* Sequential Organ Failure Assessment, *WBC* white blood cell

### Risk factors for hypothermic sepsis

Multivariable analysis was performed to determine whether patient factors were independently associated with hypothermia. The initial model contained age, BMI, cerebrovascular disease, chronic cardiovascular insufficiency, hypertension, chronic renal insufficiency, site of infection and admission origin (Additional file 1: Table S2). Interestingly, hypertension (adjusted odds ratio (aOR) 1.98, 95 % CI 1.30–3.02) and chronic cardiovascular insufficiency (aOR 3.27, 95 % CI 1.25–8.50) were associated with hypothermia. BMI was inversely correlated with hypothermia (aOR 0.96, 95 % CI 0.93–0.99). Age was not independently associated with hypothermia (aOR 1.01, 95 % CI 0.999–1.03).

### Hypothermia on admission is associated with increased mortality

**Fig. 1:**
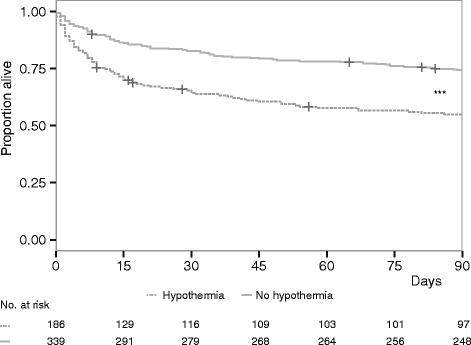
Survival curve in patients with and without hypothermia during the first 24 h of ICU admission. Kaplan–Meier plot of survival time up to 90 days after ICU admission. ***P < 0.001

ICU and hospital mortality were significantly higher in septic patients with hypothermia (Additional file 1: Table S3). There were differences in mortality at 30, 60 and 90 days and at 1 year after ICU admission (Fig. 1 and Additional file 1: Table S3). There was an increased incidence of AKI during admission in patients with hypothermia. There was no difference in the incidence of ICU-acquired infections. Multivariable logistic regression including APACHE IV scores was performed to determine if hypothermia was independently associated with mortality. Site of infection was a confounder in our study and thus was retained in the model (Additional file 1: Table S4). Hypothermia was independently associated with an increased risk of death at 90 days (aOR 2.08, 95 % CI 1.38–3.16).

### Hypothermic sepsis is not associated with altered anti-inflammatory or proinflammatory cytokine plasma levels

**Fig. 2:**
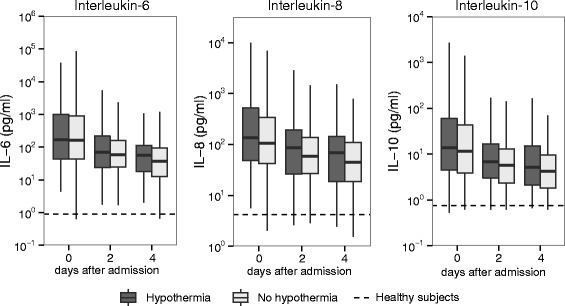
Plasma cytokine levels in patients with sepsis, stratified according to the presence of hypothermia. Box and whisker diagrams depict the median and lower quartile, upper quartile and respective 1.5 IQR as *whiskers. Dashed lines* represent median levels in healthy volunteers. Differences between patient groups were not significant

Levels of IL-13 were undetectable or low in the majority of patients and were not different between groups. Levels of IL-10 were increased in patients with sepsis compared to healthy subjects; however, there was no association with the presence of hypothermia (Fig. 2).

The proinflammatory cytokines TNF-α and IL-1β were also undetectable or low in the majority of patients and were not different between groups. IL-6 and IL-8 levels were increased in patients with sepsis; however, there was also no association with the presence of hypothermia (Fig. 2).

### Hypothermia does not affect leukocyte responsiveness upon ex vivo stimulation

**Fig. 3:**
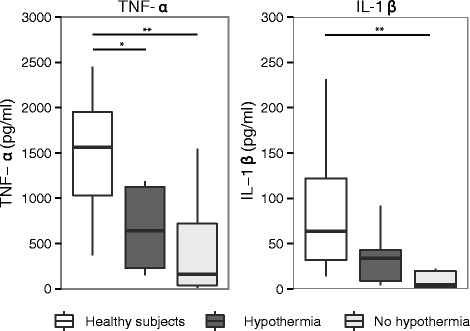
Whole blood leukocyte responsiveness to lipopolysaccharide (LPS) stratified according to the presence of hypothermia. Responsiveness of whole blood leukocytes to LPS was reduced compared to healthy subjects (*n* = 18), but was not different between hypothermic (*n* = 5) and nonhypothermic (*n* = 10) patients with sepsis. Box and whisker diagrams depict the median and lower quartile, upper quartile, and their respective 1.5 IQR as *whiskers*. *P < 0.05, **P <0.01

As hypothermia has been postulated to be an early clinical predictor of sepsis-induced immunosuppression [10], we investigated the association between hypothermia and the responsiveness of circulating immune effector cells to LPS, a marker of sepsis-induced immunosuppression [22]. Whole blood from 15 patients with sepsis, of whom 5 had hypothermia, was stimulated ex vivo and compared with blood from 18 healthy age- and gender-matched volunteers. Clinical characteristics of patients with sepsis are displayed in Additional file 1: Table S5. Patients with sepsis had a reduced capacity to release TNF-α and IL-1β upon LPS stimulation compared to healthy controls (Fig. 3). However, no differences in cellular responsiveness were observed between hypothermic and nonhypothermic patients with sepsis.

### Hypothermia is associated with increased plasma levels of the endothelial cell activation marker fractalkine

**Fig. 4:**
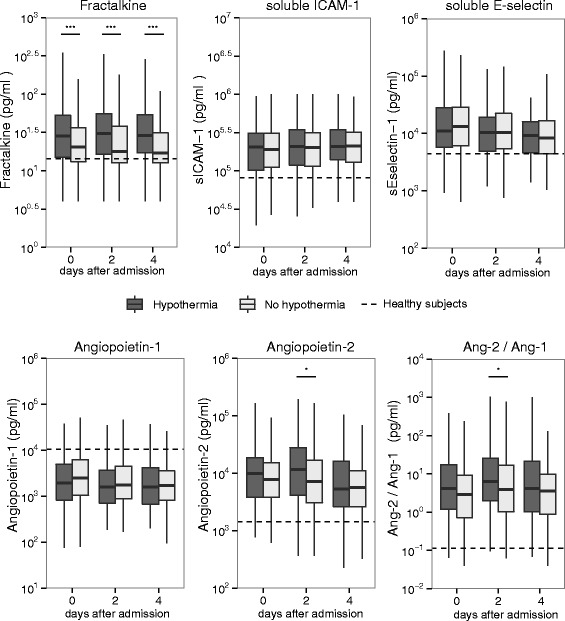
Endothelial cell activation in patients with sepsis, stratified according to the presence of hypothermia. Box-and-whisker diagrams depict the median and lower quartile, upper quartile and their respective 1.5 IQR as *whiskers. Dashed lines* represent the median in 27 healthy volunteers. *ICAM-1* intercellular adhesion molecule-1. Note: soluble ICAM-1 is also derived from leukocytes. ***P < 0.001, *P < 0.05

All markers of endothelial dysfunction were higher in patients with sepsis compared to healthy controls, except angiopoetin-1, which was lower (Fig. 4). Angiopoietin-2 and the ratio of angiopoietin-2/angiopoietin-1 were higher in hypothermic patients on day 2 compared to nonhypothermic patients with sepsis. This difference was not present at the other time points. Strikingly, levels of fractalkine, an endothelial-cell-derived chemokine, were substantially higher in hypothermic versus nonhypothermic patients on the day of admission (Fig. 4). These differences persisted on days 2 and 4 after admission.

To determine whether the higher levels of fractalkine in the hypothermic group were due to differences in disease severity, every hypothermic patient was matched to a nonhypothermic patient with a comparable APACHE IV score. Patient characteristics of the matched cohort are shown in Additional file 1: Table S6. In the subsequent analysis fractalkine remained significantly higher in patients with hypothermia (median on admission 28.5 pg/mL vs 20.8 pg/mL, *P* = 0.005, median on day 2 18.6 vs 30.8 pg/mL, *P* = 0.001 and median on day 4 17.1 vs 29.0 pg/mL, *P* = 0.001), whereas the other host response biomarkers, including angiopoietin-2 and the ratio of angiopoeietin-2/angiopoietin-1, were not different between groups. There were no differences in soluble ICAM-1, soluble E-selectin or angiopoietin-1 between hypothermic and nonhypothermic patients.

## Discussion

Hypothermia at ICU admission is independently associated with adverse outcome in patients with sepsis. In this extensive evaluation of the immune response in hypothermic sepsis, the host immune response was not altered in patients with hypothermia compared to nonhypothermic patients. The endothelial activation marker fractalkine was persistently higher in hypothermic sepsis, irrespective of disease severity. In addition, low BMI, hypertension and cardiovascular insufficiency were identified as risk factors for hypothermic sepsis. Taken together, these data suggest that vascular dysfunction could play a role in hypothermic sepsis.

An excessive anti-inflammatory response has been proposed as a mechanism for hypothermia [9]. In line with this, a recent study showed increased immunosuppression in hypothermic patients [10], thereby potentially accounting for the association with adverse outcome [3–5, 10]. In contrast, we found no difference in either proinflammatory or anti-inflammatory cytokines between hypothermic and nonhypothermic patients, even after correction for disease severity. These data are in line with a study in hypothermic patients showing no difference in circulating levels of proinflammatory cytokines [5] and extend these data by showing that levels of anti-inflammatory cytokines are also not affected by hypothermia. Moreover, whole blood stimulations resulted in similar cytokine release in hypothermic vs nonhypothermic patients. Therefore a mechanism for hypothermia directly involving anti-inflammatory cytokines seems unlikely. Rather, these data suggest that hypothermic patients do not suffer from increased immunosuppression. In support of this, the incidence of ICU-acquired infections was similar between groups, as found before in sepsis [23]. Of note, according to standard cell stimulation protocols experiments were performed ex vivo at an incubation temperature of 37 °C. If whole blood stimulations had been performed at a lower temperature to simulate the temperature of hypothermic patients, one could expect that overall the reactivity of cells in terms of cytokine production might have been slightly higher [24].

In this systematic study of risk factors for hypothermia during sepsis we identified several interesting associations. BMI was inversely correlated with hypothermia. A physiological explanation is that increased body mass likely slows the dissipation of heat from the body. As low BMI has been associated with poor outcome in the ICU [25], the relationship between hypothermia and BMI and their combined role on outcome is unclear. Leptin, released from adipose tissue, has anti-inflammatory properties and may also mediate the hypothermic response, providing a possible link between the two [26].

Of interest, we also identified hypertension and chronic cardiovascular insufficiency as risk factors. Patients with cardiovascular disease may be hampered in raising or maintaining core temperature by a dysfunction in autonomic mechanisms such as increased heart rate and blood pressure, and by shifting capillary blood flow from cutaneous to deep vascular beds [27]. Alternatively, the association between hypothermia and cardiovascular conditions may reflect the importance of an intact endothelial function in maintaining body temperature during sepsis.

Interestingly, systemic fractalkine levels were significantly higher in hypothermic patients compared to nonhypothermic patients, and this difference was maintained after correcting for disease severity. Fractalkine is a chemokine that has been implicated as a mediator in a diverse spectrum of inflammatory conditions [28]. In critically ill patients with sepsis, increased fractalkine is associated with adverse outcome [28]. Arterial and capillary endothelial cells have been identified as an important source of fractalkine during endotoxemia [29]. Also, levels of angiopoetin-2 and the ratio of angiopoietin-2/angiopoietin-1, which indicate impaired vascular integrity, were increased in the hypothermic patients compared to the nonhypothermic patients, albeit transiently. Taken together with the presence of mainly cardiovascular risk factors in patients with hypothermia, our data suggest that the endothelium may somehow be implicated in hypothermia through an as yet unknown mechanism. Although the current data do not establish a causal link between fractalkine and hypothermia, and the association between hypothermia and fractalkine in a population with significantly increased disease severity warrants further validation, the increased levels of fractalkine in hypothermia are intriguing and a detailed study of the role of the endothelium, in particular fractalkine, is warranted.

There are several shortcomings to this study. First, the timing and method of temperature measurement was not standardized. Although this could have led to increased variability in this study, we believe this effect will be limited due to the fact that core temperature measurements are standard practice in our ICUs. Second, blood sampling did not exactly coincide with timing of the temperature measurement. Although this might have diluted the results, a single instance of hypothermic temperature within 24 h significantly increases mortality, and we feel that the blood sampling does not necessarily need to be at exactly the same time as the hypothermic measurement to characterize this group. Third, results from this study are not applicable to all patients with sepsis, as we excluded patients with decreased ability to mount an adequate host response (those on steroids or those with immunodeficiency) and patients at risk of iatrogenic hypothermia (patients admitted directly from the OR). Multiple testing can cause confounding. However after Bonferroni correction fractalkine remained significantly associated with hypothermia. Also, as fractalkine was significantly elevated at all time points and remained significant after matching for disease severity, we consider these results to be valid. Last, this is an observational study and cause–effect relationships cannot be established due to the nature of this study design.

## Conclusions

In conclusion, hypothermia during sepsis is independently associated with 90-day mortality. However, neither the etiology of hypothermia or increased mortality due to hypothermia are explained by a dysfunctional hostimmune response. Low BMI, hypertension and chronic cardiovascular insufficiency are risk factors for hypothermic sepsis. Hypothermia is associated with increased levels of the endothelial-derived biomarker fractalkine. The functional role of fractalkine and the endothelium in the context of hypothermic sepsis requires further study.

## Additional file

Additional file 1: Figure S1. Selection of study patients. **Table S1.** Causative pathogens. **Table S2.** Multivariable logistic regression analysis to identify risk factors for hypothermia. **Table S3.** Outcomes of sepsis patients with and without hypothermia during the first 24 h of admission. **Table S4.** Association between hypothermia and 90-day mortality in patients with sepsis, adjusted for confounders. **Table S5.** Clinical characteristics of sepsis patients included in ex vivo whole blood stimulation analysis. **Table S6.** Clinical characteristics of sepsis patients with and without hypothermia of admission matched for APACHE IV score. (DOC 213 kb)

## Abbreviations

AIC: Akaike information criterion
AKI: acute kidney injury
ALI: acute lung injury
aOR: adjusted odds ratio
APACHE: Acute Physiology and Chronic Health Evaluation
BMI: body mass index
CI: confidence interval
ICAM: intercellular adhesion molecule
ICU: intensive care unit
IL: interleukin
LPS: lipopolysaccharide
OR: operating room
SOFA: Sequential Organ Failure Assessment
TNF: tumor necrosis factor
