# Supplementary material for: Risk factors, host response and outcome of hypothermic sepsis
Source: Crit Care. 2016 Oct 14;20:328. doi: 10.1186/s13054-016-1510-3 (PMC5064908; doi:10.1186/s13054-016-1510-3)
Supplement: Additional file 1: Figure S1. — Selection of study patients. Table S1. Causative pathogens. Table S2. Multivariable logistic regression analysis to identify risk factors for hypothermia. Table S3. Outcomes of sepsis patients with and without hypothermia during the first 24 h of admission. Table S4. Association between hypothermia and 90-day mortality in patients with sepsis, adjusted for confounders. Table S5. Clinical characteristics of sepsis patients included in ex vivo whole blood stimulation analysis. Table S6. Clinical characteristics of sepsis patients with and without hypothermia of admission matched for APACHE IV score. (DOC 213 kb) [file 13054_2016_1510_MOESM1_ESM.doc]

SUPPLEMENTAL MATERIAL

**Risk factors, host response and outcome of hypothermic sepsis**

Maryse A. Wiewel, MD^*^, Matthew B.A. Harmon, MD^*^, Lonneke A. van Vught, MD, Brendon P. Scicluna, PhD, Arie J. Hoogendijk, PhD, Janneke Horn, MD, PhD, Aeilko H. Zwinderman, PhD^3^, Olaf L. Cremer, MD, PhD, Marc J. Bonten, MD, PhD, Marcus J. Schultz, MD, PhD, Tom van der Poll, MD, PhD, Nicole P. Juffermans, MD, PhD^*^, W. Joost Wiersinga, MD, PhD^*^

* Contributed to this article equally

**Additional file 1: Figure S1.** Selection of study patients.

**Additional file 1: Table S1. Causative pathogens**

|  | Hypothermia | No hypothermia | *p* |
| --- | --- | --- | --- |
|  | N = 186 | N = 339 |  |
| Gram-positive bacteria (%) | 90 (48.4) | 162 (47.8) | .82 |
| Gram-negative bacteria (%) | 123 (66.1) | 220 (64.9) | .73 |
| Yeast/fungi (%) | 13 (7) | 37 (10.9) | .18 |
| Other (%) | 26 (14) | 34 (10) | .19 |
| Unknown (%) | 25 (13.4) | 60 (17.7) | .28 |

Percentages represent the pathogens divided by the number of patients. In some cases

multiple causative pathogens were isolated.

**Additional file 1: Table S2**. **Multivariable logistic regression analysis to identify risk factors for**

**hypothermia**

|  | OR | 95% C | *p* |
| --- | --- | --- | --- |
| Age | 1.01 | 0.999-1.03 | .07 |
| Body mass index | 0.96 | 0.93-0.99 | .009 |
| Hypertension | 1.98 | 1.30-3.02 | .001 |
| Chronic cardiovascular insufficiency | 3.27 | 1.25-8.50 | .02 |

**Additional file 1: Table S3. Outcomes of sepsis patients with and without hypothermia during the first 24 hours of admission**

|  | Hypothermia | No hypothermia | *p* |
| --- | --- | --- | --- |
|  | N = 186 | N = 339 |  |
| Acute kidney injury (%) | 105 (56.5) | 137 (40.4) | .002 |
| Renal replacement therapy (%) | 40 (21.5) | 41 (12.1) | .005 |
| Acute lung injury (%) | 25 (31.2) | 113 (33.3) | .62 |
| ICU-acquired weakness (%) | 13 (7) | 20 (5.9) | .72 |
| ICU-acquired infections (%) | 15 (8.1) | 24 (7.1) | .72 |
| ICU-mortality (%) | 48 (25.8) | 38 (11.2) | <.001 |
| Hospital mortality (%) | 69 (37.1) | 69 (20.4) | <.001 |
| 30 day mortality (%) | 66 (35.5) | 59 (17.4) | <.001 |
| 60 day mortality (%) | 78 (41.9) | 74 (21.8) | <.001 |
| 90 day mortality (%) | 84 (45.2) | 87 (25.7) | <.001 |
| 1 year mortality (%) | 104 (55.9) | 122 (36) | <.001 |

**Additional file 1: Table S4. Association between hypothermia and 90-day mortality in patients with sepsis,**

adjusted for confounders

|  | OR | 95% CI | *p* |
| --- | --- | --- | --- |
| Hypothermia | 2.47 | 1.69 – 3.61 | <.0001 |
| Hypothermia + APACHE IV score ^a^ | 1.91 | 1.28 – 2.87 | .0017 |
| Hypothermia + APACHE IV score ^a^ + Site of infection | 2.08 | 1.38 – 3.16 | .0005 |

APACHE, acute physiology and chronic health evaluation.

^a^ Temperature not included in score

**Additional file 1: Table S5. Clinical characteristics of sepsis patients included in ex vivo whole blood stimulation analysis.**

|  | Hypothermia | No hypothermia |
| --- | --- | --- |
|  | N = 5 | N = 10 |
| **Demographics** |  |  |
| Age, years, mean [SD] | 61.4 [12.9] | 67.1 [13.7] |
| Gender, male (%) | 1 (20) | 6 (60) |
| BMI, kg/m2, mean [SD] | 29.1 [9.8] | 29.3 [8.9] |
| Charlson score, median [IQR] | 3 [0-3] | 2 [0-3] |
| **Site of infection** |  |  |
| Pulmonary (%) | 2 (40) | 6 (60) |
| Abdominal (%) | 0 (0) | 1 (10) |
| Urinary tract (%) | 2 (40) | 2 (20) |
| Other (%) | 5 (100) | 10 (100) |
| Co-infection (%) | 1 (20) | 1 (10) |
| **Severity of disease first 24h** |  |  |
| Mean temperature first 6 h, median [IQR] | 36.1 [35-36.9] | 37.1 [36.9-38] |
| Mean temperature first 24 h, median [IQR] | 36 [35.6-36.3] | 37.4 [37.2-37.8] |
| APACHE IV score, median [IQR] ^a^ | 75 [65-113] | 73 [63-80] |
| SOFA score, median [IQR] ^b^ | 9 [8-9] | 7.5 [6-9] |
| Acute kidney injury (%) | 3 (60) | 4 (40) |
| Acute lung injury (%) | 2 (40) | 4 (40) |
| Shock (%) | 1 (20) | 4 (40) |
| **Clinical laboratory parameters first 24h** | |  |
| WBC count max.( x10^9/l), median [IQR] | 13.1 [8.3-13.5] | 12.2 [10.5-18.8] |
| WBC count min.( x10^9/l), median [IQR] | 9.4 [7-12.7] | 9.2 [7.8-15.2] |
| Platelets min. (x10^9/l), median [IQR] | 109 [72-276] | 158 [138-199] |
| Lactate max. (mmol/l), median [IQR] | 1.1 [1.1-1.9] | 2.4 [2.124] |
| Prothrombin time max. (s), median [IQR] | 16 [13.5-16.7] | 16.8 [12.1-19.9] |
| Creatinin max. (μmol/l), median [IQR] | 257 [200-421] | 113 [88-142] |
| C-reactive protein (mg/l), median [IQR] | 160 [128-219] | 128 [71-177] |
| **Outcome** |  |  |
| ICU-mortality (%) | 1 (20) | 1 (10) |
| Hospital mortality (%) | 2 (40) | 4 (40) |
| 30 day mortality (%) | 2 (40) | 2 (20) |
| 60 day mortality (%) | 3 (60) | 3 (30) |
| 90 day mortality (%) | 3 (60) | 4 (40) |
| 1 year mortality (%) | 3 (60) | 4 (40) |

APACHE, acute physiology and chronic health evaluation; IQR, interquartile range; SD, standard deviation; SOFA, sequential organ failure

assessment; WBC, white blood cell.

^a^ Temperature not included in score

^b^ Central nervous system not included in score due to large number of sedated patients

**Additional file 1: Table S6. Clinical characteristics of sepsis patients with and without hypothermia of admission matched for APACHE IV score.**

|  | Hypothermia | No hypothermia | *p* |
| --- | --- | --- | --- |
|  | N = 186 | N = 186 |  |
| **Demographics** |  |  |  |
| Age, years, mean [SD] | 65.0 [13.8] | 62.1 [15.9] | .06 |
| Gender, male (%) | 114 (61.3) | 114 (61.3) | >.99 |
| BMI, kg/m2, mean [SD] | 25.6 [5.7] | 27.1 [6.5] | .02 |
| Charlson score, median [IQR] | 5 [3-6] | 4 [2-6] | .20 |
| **Site of infection** |  |  |  |
| Pulmonary (%) | 79 (42.5) | 81 (43.5) | .91 |
| Abdominal (%) | 29 (15.6) | 36 (19.4) | .40 |
| Urinary tract (%) | 32 (17.2) | 21 (11.3) | .13 |
| Other (%) | 18 (9.7) | 26 (14) | .27 |
| Co-infection (%) | 28 (15.1) | 22 (11.8) | .43 |
| **Severity of disease first 24h** |  |  |  |
| Mean temperature first 6 h, median [IQR] | 36.1 [35.4-37] | 37.1 [36.5-37.9] | <.0001 |
| Mean temperature first 24 h, median [IQR] | 36.3 [35.8-36.9] | 37.3 [36.7-37.8] | <.0001 |
| APACHE IV score, median [IQR] ^a^ | 82 [67-103] | 82 [67-100] | .54 |
| SOFA score, median [IQR] ^b^ | 8 [5-10] | 7 [4-9] | .008 |
| Acute kidney injury (%) | 92 (49.5) | 72 (38.7) | .06 |
| Acute lung injury (%) | 49 (26.3) | 51 (27.4) | .90 |
| Shock (%) | 74 (39.8) | 62 (33.3) | .24 |
| **Clinical laboratory parameters first 24h** | |  |  |
| WBC count max.( x10^9/l), median [IQR] | 16.1 [10.9-25.5] | 14.9 [10-19.4] | .07 |
| WBC count min.( x10^9/l), median [IQR] | 12.6 [7.1-19.1] | 12.7 [8-16.5] | .47 |
| Platelets min. (x10^9/l), median [IQR] | 189 [120-264] | 200 [130-275] | .54 |
| Lactate max. (mmol/l), median [IQR] | 3.2 [1.6-6.5] | 2.7 [1.6-5.1] | .16 |
| Prothrombin time max. (s), median [IQR] | 16.5 [14.1-20.7] | 15.6 [13.2-19.2] | .03 |
| Creatinin max. (μmol/l), median [IQR] | 121 [80-209] | 103 [73-174] | .03 |
| C-reactive protein (mg/l), median [IQR] | 146 [82-258] | 154 [93-248] | .81 |
| **Outcome** |  |  |  |
| ICU-mortality (%) | 48 (25.8) | 26 (14) | .008 |
| Hospital mortality (%) | 69 (37.1) | 47 (25.3) | .01 |
| 30 day mortality (%) | 66 (35.5) | 41 (22) | .003 |
| 60 day mortality (%) | 78 (41.9) | 49 (26.3) | .0005 |
| 90 day mortality (%) | 84 (45.2) | 61 (32.8) | .01 |
| 1 year mortality (%) | 104 (55.9) | 79 (42.5) | .007 |

APACHE, acute physiology and chronic health evaluation; IQR, interquartile range; SD, standard deviation; SOFA, sequential organ failure assessment; WBC, white blood cell.

^a^ Temperature not included in score

^b^ Central nervous system not included in score due to large number of sedated patients
